# Hospital acquired infection prevalence survey: is there any Franc-Comtois paradox?

**DOI:** 10.1186/2047-2994-4-S1-P278

**Published:** 2015-06-16

**Authors:** N Floret, L Paulet, A-L Parmentier, F Mauny

**Affiliations:** 1 Réseau Franc-Comtois de Lutte Contre les Infections Nosocomiales, Centre Hosptialier Universitaire, Besançon, France; 2 Centre de Méthodologie Clinique, Centre Hosptialier Universitaire, Besançon, France

## Introduction

Hospital acquired infection (HAI) prevalence surveys have been conducted in French healthcare facilities (HCF) since 1996. Overall HAI prevalence rate remained steady around 5.0% in France from 2006 to 2012. A decreased by 10.8% was found when a multilevel analysis was applied and contrasting trends were shown: no significant change for short stay hospitalization (from 5.3% to 5.6%) whereas a significant decrease by 21% was observed in the others. In Franche-Comté (FC), from 2006 to 2012, an unexpected increasing HAI prevalence trend by 17.3% (from 5.2% to 6.1%) was observed.

## Objectives

To assess if the FC HAI prevalence trend is in line with the national trend.

## Methods

All adult patients in the 10 FC HCF that have participated every year from 2006 to 2013 were enrolled. The outcome was patient infected status. Sets of confounding factors were considered at 2 levels: patient and HCF. Almost factors were dichotomous: sex, immune-compromised status, exposure to invasive device and to surgical procedure, type of stay. Dummy variables were generated for ordinal variables (age, Mac Cabe status, hospital size) taking the lowest category as the reference group and year as a continuous variable. Multilevel analysis was applied using the Poisson regression. Statistical analysis was performed using MlWin software.

## Results

From 2006 to 2013, 20,629 patients were enrolled. The HAI prevalence rate increased from 4.5% to 6.3% and a crude linear time trend was statistically significant (RR=1.03 _95_CI[1.01 – 1.06], p=0.01). For short stay, the linear time trend wasn’t statistically significant (RR=0.98 _95_CI[0.91 – 1.06], p=0.59). To be exposed to invasive device (RR=3.65 _95_CI[2.44 – 5.47], p<10^-3^) and to have been exposed to surgical procedure (RR=1.77 _95_CI[1.03 – 3.05], p=0.04) were the only statistically significant risk factors. By contrast, in other type of stay, all studied factors were statistically significant as the linear trend also (RR=1.03 _95_CI[1.00 – 1.06], p=0.05).

## Conclusion

FC HAI prevalence trend doesn’t match the national one. The crude linear time trend was statistically significant. To be hospitalized in short stay was associated to a lower risk of HAI whereas to be hospitalized in other type stay was at higher risk.

## Disclosure of interest

None declared.

